# Subdomain Adaptation Capsule Network for Partial Discharge Diagnosis in Gas-Insulated Switchgear

**DOI:** 10.3390/e25050809

**Published:** 2023-05-17

**Authors:** Yanze Wu, Jing Yan, Zhuofan Xu, Guoqing Sui, Meirong Qi, Yingsan Geng, Jianhua Wang

**Affiliations:** State Key Laboratory of Electrical Insulation and Power Equipment, Xi’an Jiaotong University, Xi’an 710049, China; yzwu199912@stu.xjtu.edu.cn (Y.W.); xzf200011@stu.xjtu.edu.cn (Z.X.); guoqingsui@stu.xjtu.edu.cn (G.S.); mrqi200010@stu.xjtu.edu.cn (M.Q.); ysgeng@mail.xjtu.edu.cn (Y.G.); jhwang@mail.xjtu.edu.cn (J.W.)

**Keywords:** partial discharge, capsule network, subdomain adaptation, gas-insulated switchgear, fault diagnosis

## Abstract

Deep learning methods, especially convolutional neural networks (CNNs), have achieved good results in the partial discharge (PD) diagnosis of gas-insulated switchgear (GIS) in the laboratory. However, the relationship of features ignored in CNNs and the heavy dependance on the amount of sample data make it difficult for the model developed in the laboratory to achieve high-precision, robust diagnosis of PD in the field. To solve these problems, a subdomain adaptation capsule network (SACN) is adopted for PD diagnosis in GIS. First, the feature information is effectively extracted by using a capsule network, which improves feature representation. Then, subdomain adaptation transfer learning is used to accomplish high diagnosis performance on the field data, which alleviates the confusion of different subdomains and matches the local distribution at the subdomain level. Experimental results demonstrate that the accuracy of the SACN in this study reaches 93.75% on the field data. The SACN has better performance than traditional deep learning methods, indicating that the SACN has potential application value in PD diagnosis of GIS.

## 1. Introduction

Gas-insulated switchgear (GIS) is widely used in the power grid because of its advantages of good insulation, high reliability, and small footprint [1]. However, the failure rate of GIS is much higher than that stipulated by the International Electro Technical Commission standard, which seriously affects power supply reliability. Insulation defects in GIS are one of the significant causes of GIS failure, leading to huge loss to the power grid. As a prominent sign of an insulation defect, partial discharge (PD) may result in the insulation failure of GIS. Therefore, performing PD diagnosis of GIS is essential for discovering insulation defects early and removing them effectively, which is crucial to ensure reliable operation of the power system. 

Currently, GIS PD diagnosis methods can be divided into model-driven and data-driven methods. Data-driven methods have become a popular research area because they address the difficulty of finding or building models that fit data, which comprise machine learning (ML) and deep learning (DL). ML methods of PD diagnosis consist of two parts: Feature extraction and PD type classification. Feature extraction uses signal processing technology, such as wavelet packet decomposition [2] and the short-time Fourier transform [3], to denoise and extract representative features. PD type classification utilizes different classification methods such as support vector machines [4] and K-nearest neighbor [5] and random forest [6] approaches. However, although manual feature extraction in ML methods seriously relies on expert experience, the performance of the classifier is greatly affected by the feature and generalization ability of the ML model; thus, there are great discrepancies among different classifiers under different states.

With the rapid development of artificial intelligence, DL, especially using convolutional neural networks (CNNs), has received wide attention because of its powerful capability of feature extraction and classification. Song et al. [7] employed a deep CNN to recognize PD patterns under various data sources and improved the recognition accuracy compared with traditional ML methods. Wang et al. [8] proposed a light-scale CNN for PD pattern recognition and verified the superiority of the light-scale CNN on the recognition accuracy and calculation time. Liu et al. [9] adopted a CNN with a long short-term memory model for distinguishing PD types, achieving greater accuracy than that of other traditional analysis methods. However, the CNN needs to learn features of PD from massive samples, and the diagnosis capability of the model seriously degrades when the sample size is reduced.

To solve the problem of low accuracy under small-sample conditions, deep transfer learning (DTL) has been continuously studied in recent years. Among the many DTL methods, domain adaptation based on maximum mean discrepancy (MMD) [10] is studied as the most popular method, as it has a flexible loss function and involves an uncomplicated training process. Guo et al. [10] adopted deep convolutional transfer learning to accomplish fault diagnosis with various data sources from different machines; their approach employs a condition recognition module and uses MMD as the domain loss. Zhu et al. [11] presented a DTL-based convolutional network for fault diagnosis in different working conditions in which Gaussian kernels were added for MMD calculation optimization. Their model performance was validated by experiments and compared with shallow learning methods. However, MMD domain adaptation mainly learns the global distribution of source and target domains, ignoring the confusion between subdomains for each PD type of GIS.

To compensate for the deficiency of MMD domain adaptation, subdomain adaptation was proposed to learn the local domain distribution. Tian et al. [12] proposed a multi-source subdomain adaptation transfer learning method to improve the generalization ability of diagnostic models. Extensive experiments demonstrated that their proposed model has significant advantages in cross-domain fault diagnosis. Zhu et al. [13] proposed a simulation-data-driven subdomain adaptation adversarial transfer learning network that combines adversarial learning and subdomain adaptation and verified its effectiveness in rolling bearing fault diagnosis. Wang et al. [14] used a novel subdomain adaptation transfer learning network for the fault diagnosis of roller bearings and tested its superiority with six transfer tasks. 

However, the feature classifiers of the above methods are mostly based on CNNs, which ignores the relationship between features because of the scalar form of the full connected layer, which can lead to feature information loss and limited diagnostic accuracy of PD in GIS. Therefore, the capsule network (CapsNet) [15] was proposed, which considers the relationship between features in feature extraction and has the ability to fit complex data features. CapsNet effectively improves diagnostic accuracy and has achieved excellent results in many fields. Chen et al. [16] adopted CapsNet to realize the fault recognition of high-speed train bogies under various working conditions and proved its efficiency through an experimental comparison with a CNN. Ke et al. [17] proposed a compound fault diagnosis method based on CapsNet for a modular multilevel converter, verifying it to have excellent fault recognition accuracy. Wang et al. [18] used CapsNet for fault classification and enhanced diagnostic performance through adversarial training. The accuracy of their proposed method is higher than that of other advanced methods. 

Inspired by adaptive and capsule networks, we propose a subdomain adaptation capsule network (SACN) for on-site small-sample GIS PD diagnosis. First, an improved CapsNet is proposed to enhance the extraction capability and reduce information loss. Then, an adaptative local maximum mean discrepancy (ALMMD) of subdomain adaptation is adopted to measure the distance between subdomains adaptively and restrain the negative effect of the category discrepancy of the samples. Finally, the model is applied to PD diagnosis under the small-sample condition on site. The main contributions of this study are generalized as follows:

- A SACN is proposed for small-sample GIS PD diagnosis in the field. To the best of our knowledge, this is the first time that SACN has been applied to GIS PD diagnosis.
- A novel method of subdomain adaptation is introduced into GIS PD diagnosis. ALMMD is used as the distance criterion of subdomain adaptation to calculate the distance between subdomains adaptively and solves the problems of local information ignored by the MMD domain adaptation.
- An improved CapsNet is introduced into the feature extraction to further improve feature extraction capability. A self-routing algorithm is introduced into CapsNet to improve the routing coefficient generation strategy, thereby improving the computational efficiency and classification accuracy of CapsNet.
- Laboratory and field experiments are constructed to verify the superiority of the SACN proposed in this study. The experimental results show that the model proposed has better performance than traditional DL methods in on-site small-sample GIS PD diagnosis.

## 2. Preliminaries

### 2.1. Domain Adaptation

Domain adaptation is one of the typical algorithms employed in DTL [15]. Domain adaptation aims to obtain the common features of source and target domains when the learning task is the same. Under its theory, the source domain $D_{s}={\{\left(x_{i}^{s},y_{i}^{s}\right)\}}_{i=1}^{n_{s}}$ conforms to the distribution of *p* and the target domain $D_{t}={\{x_{j}^{t},y_{j}^{t}\}}_{j=1}^{n_{t}}$ conforms to the distribution of *q*. *D_s_* consists of *n_s_* samples, including input *x*^s^ and label vector *y*^s^, while *D*_t_ includes *n*_t_ samples. To establish the specific character of the GIS fault diagnosis field, the source domain is designed as the abundant data from the laboratory while the target domain is from the field. The kernel of domain adaptation establishes a model of DL to transfer distribution characteristics and promote the precision of classification of the target domain in the case of insufficient data support. The optimization process obeys the principle of minimizing the classification loss and the discrepancy between training and test sets. According to the proposed principle, the optimization objective function can be expressed as

(1) $$\underset{f}{\min}\frac{1}{n_{s}}\sum_{i=1}^{n_{s}}J\left(f\left(x_{i}^{s}\right),y_{i}^{s}\right)+\alpha \overset{\wedge }{d}(p,q),$$

where J(⋅,⋅) is the cross-entropy loss function, $\overset{\wedge }{d}(\cdot ,\cdot )$ represents the loss of domain transfer, α expresses the coupling relationship as the trade-off parameter, and $f\left(x_{i}^{s}\right)$ is the classification operation of input $x_{i}^{s}$ to get close to the true label $y_{i}^{s}$.

As one of the distance criteria of domain adaptation, MMD is used most frequently. MMD maps the initial feature distribution that is indivisible linearly into the reproducing kernel Hilbert space (RKHS) to be divisible easily. The kernel function of RKHS amounts to the inner product of the mapping function. MMD mainly focuses on global distribution alignment while ignoring the feature association of different subdomains. The difference in the function means mapped with the reproducing kernel can be represented as

(2) $$d_{H}^{2}\left(D_{s},D_{t}\right)={\left\|\frac{1}{n_{s}}\sum_{i=1}^{n_{s}}\phi \left(D_{s}\right)-\frac{1}{n_{t}}\sum_{j=1}^{n_{t}}\phi \left(D_{t}\right)\right\|}_{H}^{2},$$

where *H* represents RKHS and ϕ is the mapping function.

RKHS is generated with the embedding of a kernel mean such as a Gaussian or Laplace kernel. Then, the formula via empirical estimation is:

(3) $$d_{H}^{2}\left(x_{i},x_{j}\right)={\left\|\frac{1}{n_{s}^{2}}\sum_{i=1}^{n_{s}}\sum_{j=1}^{n_{s}}k\left(x_{i}^{s},x_{j}^{s}\right)-\frac{2}{n_{s}n_{t}}\sum_{i=1}^{n_{s}}\sum_{j=1}^{n_{t}}k\left(x_{i}^{s},x_{j}^{t}\right)+\frac{1}{n_{t}^{2}}\sum_{i=1}^{n_{t}}\sum_{j=1}^{n_{t}}k\left(x_{i}^{t},x_{j}^{t}\right)\right\|}_{H},$$

where *k* is the kernel of the inner product.

### 2.2. Capsule Network

To solve the problem of feature extraction inadequacy and overfitting of the CNN, CapsNet raises the capsule structure and the feature selection method via a dynamic routing algorithm. A classical CapsNet framework is divided into three components: a one-dimensional convolutional layer, a primary capsule (PCaps) layer, and a digital capsule (DCaps) layer. The one-dimensional convolutional layer is composed of multiple convolution-pool layers. The initial features are extracted by several convolutional layers with pooling layers. In contrast to the scalar neurons in a CNN, a capsule layer contains a certain number of capsules that compose a group of vector neurons. 

**Figure 1:**
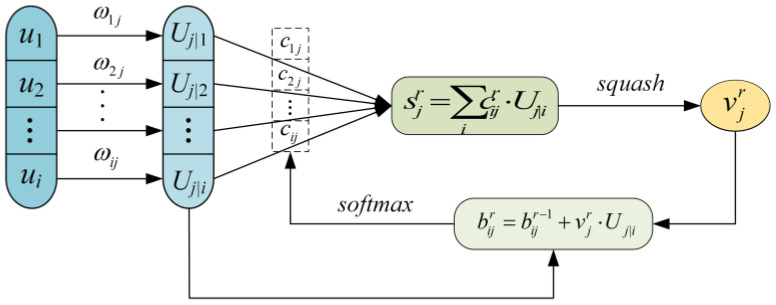
Dynamic routing algorithm.

CapsNet learns from the strength of feature extraction of the CNN. Meanwhile, CapsNet raises the capsule structure and the feature selection method via a dynamic routing algorithm. PCaps is used for describing the local feature of the object, and the purpose of DCaps is to express the abstract feature. Then, feature information from PCaps is clustered and updated into DCaps through the dynamic routing algorithm. The algorithm process is shown in Figure 1.

If *u_i_* represents the capsule in the (*j −* 1)th layer, then the prediction vector $U_{j|i}$ can be calculated as follows:

(4) $$U_{j|i}=\omega_{ij}u_{i},$$

where $\omega_{ij}$ is the affine transformation matrix as weight adding to *u_i_*. The total input vector $s_{j}$ is obtained by the weighted sum of the prediction vector as follows:

(5) $$s_{j}=\sum_{i}c_{ij}\cdot U_{j|i},$$

where *c_ij_* is the coupling parameter that satisfies $\sum c_{ij}=1$. Then, *v_j_* is designed as the output vector of the *j*th capsule calculated by the nonlinear function *squash* as:

(6) $$v_{j}=\frac{{\left\|s_{j}\right\|}^{2}}{1+{\left\|s_{j}\right\|}^{2}}\cdot \frac{s_{j}}{\left\|s_{j}\right\|}.$$

The weight parameter *c_ij_* is gained and updated iteratively as follows:

(7) $$c_{ij}=\frac{\exp(b_{ij})}{\sum_{k}\exp(b_{ik})},$$

where *b_ij_* is the logarithmic prior probability whose initial value is zero.

In the process of forward propagation, *c_ij_* is obtained using Equation (7) and *v_j_* is received according to Equations (5) and (6). *c_ij_* is updated and modified utilizing the iteration of *b_ij_*, and *b_ij_* is from the change in *v_j_*. Then, *s_j_* is further corrected by forward propagation to gain the output vector *v_j_*. The coupling coefficients above can be acquired and optimized by the iteration of dynamic routing [19].

## 3. Proposed Method

**Figure 2:**
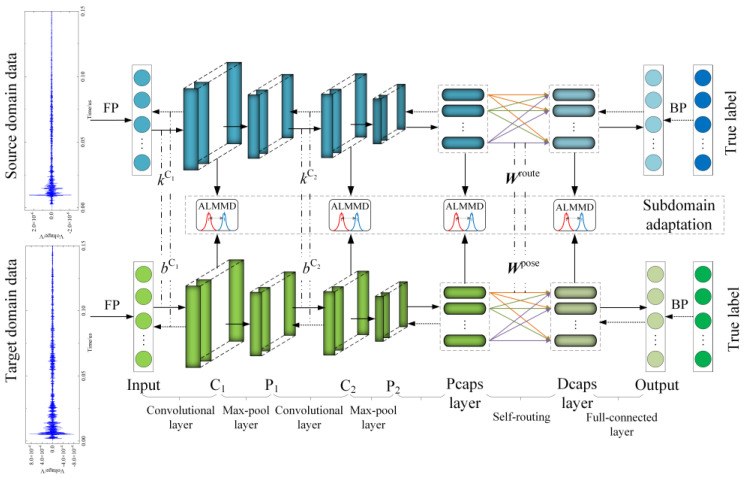
Structure of SACN.

In this study, we propose a SACN for on-site small-sample PD diagnosis in GIS. The overall architecture of our SACN is shown as Figure 2; it is composed of three parts: a feature extractor, subdomain adaptation, and a classifier. The feature extractor adopts CapsNet with a self-routing algorithm to simplify the complex iterative process of dynamic routing in the traditional CapsNet. In the subdomain adaptation, ALMMD is utilized in the computation of the domain loss function to reduce the confusion of different subdomains and narrow the local distribution of source and target domains. Compared with domain adaptation, subdomain adaptation not only guarantees the largest distance between classes but also ensures the smallest distance between samples in the same class, thus avoiding the boundary confusion between different classes. The classifier is used to determine the category of GIS PD, and the domain-aligned and matched features are used as input to realize small-sample PD diagnosis in the field.

### 3.1. Feature Extractor

In this study, capsule networks are used to extract discriminative features in GIS PD diagnosis. Because the dynamic routing algorithm used in the traditional CapsNet employs a complex iteration mechanism, which brings a huge computation burden when the input space dimension is large, a self-routing capsule network (SR-CapsNet) [20] is proposed. Instead of dynamic routing, the self-routing algorithm between the capsule layers can process lower capsules of different scales with a much lower calculation cost and fewer model parameters because of its non-iteration characteristic. 

The self-routing algorithm introduces two learnable weight matrices: a routing weight matrix and a pose weight matrix.

The routing weight matrix $W^{\mathrm{route}}$ is used to calculate the routing coefficient *c_ij_*, which indicates the probability that the upper capsule is activated. The routing coefficient is calculated as follows:

(8) $$c_{ij}=\mathrm{softmax}{\left(W_{i}^{\mathrm{route}}u_{i}\right)}_{j},$$

where $u_{i}$ is the capsule pose vector of the (*l−*1)th layer and softmax is the nonlinear activation function. 

The routing coefficient *c_ij_* is then multiplied by the activation scalar to acquire the activation scalar of the upper layer. The activation scalar is acquired by quantifying the initial feature to reflect the probability value of activation of the (*l−*1)th layer. The activation scalar of the *l*th layer, $a_{j}$, is generated as follows:

(9) $$a_{j}=\frac{\sum_{i\in N_{l}}c_{ij}a_{i}}{\sum_{i\in N_{l}}a_{i}},$$

where $N_{l}$ is the number of capsules in the (*l−*1)th layer.

The other learnable weight matrix of self-routing is the pose weight matrix used to generate the prediction vector, which is calculated as follows:

(10) $$u_{i|j}=W_{ij}^{\mathrm{pose}}u_{i},$$

where $u_{i|j}$ is the prediction capsule of *l*th layer that is affected by activation scalar $a_{j}$ to update the capsules in the *l*th layer:

(11) $$u_{j}=\frac{\sum_{i\in N_{l}}c_{ij}a_{i}u_{i|j}}{\sum_{i\in N_{l}}a_{i}}.$$

The convolution-pool layers in SR-CapsNet apply a multiscale convolution method to extract the multiscale features in the fault data and enrich the information of the PD diagnosis. Multiscale convolution can extract the detail via a shallower network than a deep convolution network. The process proposed is described as:

(12) $$y_{mc}=\mathrm{concentrate}\left(y_{1},\ldots ,y_{n}\right),$$

where $y_{1},\ldots ,y_{n}$ is the output of convolution kernels of various sizes and concentrate(⋅) represents the splicing in the direction of the channel. Some of the parameters of the feature extractor are shown in Table 1, where 8 × (4) × 8 represents that the vector dimension is four, and the feature layer width is eight.

### 3.2. Subdomain Adaptation

A subdomain contains different samples of the same class. To resolve boundary confusion of different subdomains caused by domain adaptation, subdomain adaptation addresses the issue of distribution alignment at the subdomain level. Therefore, it solves the problem that different categories of data are mixed together and cannot be separated accurately. Compared with MMD domain adaptation, local MMD (LMMD) obtains the distance between samples of the same type in different domains and aligns the distribution of the same category of data. However, the weight ratio of the distance of each category sample in the calculation of LMMD is the same and cannot be distinguished. Consequently, the addition of adaptive parameters improves LMMD to ALMMD, which can dynamically adjust the distance of each category sample. To calculate the distance between subdomains better and restrain the negative effect of the category discrepancy of the samples of the same type, the following ALMMD is proposed:

(13) $${\overset{\wedge }{d}}_{\mathrm{ALMMD}}={\sum_{n=1}^{N}\left\|\sum_{i}^{n_{s}}\omega_{i}^{s,n}\phi \left(z_{i,m}^{s}\right)-\sum_{j}^{n_{t}}\omega_{j}^{t,n}\phi \left(z_{j,m}^{t}\right)\right\|}_{H}^{2},$$

where $\alpha^{n}\left(n=1,2,\ldots ,N-1\right)$ is the adaptative parameter, with $\left\{\alpha^{n}\right\}$ being updated with the loss function value decreasing and promoting the capture of the domain distance dynamically and adaptively, and *N* is the number of categories. The weight of the distribution distance of features in the source domain $\omega_{i}^{s,c}$ and the weight of the target domain $\omega_{j}^{t,c}$ in the *nth* domain are calculated as:

(14) $$\omega_{i}^{n}=\frac{y_{i}^{s}}{\sum_{i}^{n_{s}}y_{i}^{s}},$$

(15) $$\omega_{j}^{n}=\frac{Cls\left(z_{j,m}^{t}\right)}{\sum_{j}^{n_{t}}Cls\left(z_{j,m}^{t}\right)}.$$

The calculation of ALMMD then proceeds as follows:

(16) $${\overset{\wedge }{d}}_{\mathrm{ALMMD}}=\sum_{n=1}^{N}\left[\begin{array}{l} \frac{1}{n_{s}^{2}}\sum_{i}^{n_{s}}\sum_{j}^{n_{s}}\omega_{i}^{s,n}\omega_{j}^{s,n}k\left(z_{i,m}^{s},z_{j,m}^{s}\right)\\ +\frac{1}{n_{t}^{2}}\sum_{i}^{n_{t}}\sum_{j}^{n_{t}}\omega_{i}^{t,n}\omega_{j}^{t,n}k\left(z_{i,m}^{t},z_{j,m}^{t}\right)\\ -\frac{2}{n_{s}n_{t}}\sum_{i}^{n_{s}}\sum_{j}^{n_{t}}\omega_{i}^{s,n}\omega_{j}^{t,n}k\left(z_{i,m}^{s},z_{j,m}^{t}\right) \end{array}\right].$$

### 3.3. Training Process

The SACN model is trained via minimizing the classification loss of source and target domains and the ALMMD loss. The loss function on the PD type classification of the source domain and the training data selected from field data can be expressed as follows:

(17) $$J_{s}=\frac{1}{n_{s}}\sum_{i=1}^{n_{s}}J\left(y_{i}^{s},f\left(x_{i}^{s}\right)\right),$$

(18) $$J_{t}=\frac{1}{n_{t\_\mathrm{part}}}\sum_{j=1}^{n_{t\_\mathrm{part}}}J\left(y_{j}^{t},f\left(x_{j}^{t}\right)\right),$$

where J(⋅,⋅) is the loss function based on cross-entropy.

The ALMMD loss function is:

(19) $$J_{\mathrm{ALMMD}}=\frac{\alpha^{n}}{N}\sum_{n=1}^{N}\left[\begin{array}{l} \frac{1}{n_{s}^{2}}\sum_{i}^{n_{s}}\sum_{j}^{n_{s}}\omega_{i}^{s,n}\omega_{j}^{s,n}k\left(z_{i,m}^{s},z_{j,m}^{s}\right)\\ +\frac{1}{n_{t}^{2}}\sum_{i}^{n_{t}}\sum_{j}^{n_{t}}\omega_{i}^{t,n}\omega_{j}^{t,n}k\left(z_{i,m}^{t},z_{j,m}^{t}\right)\\ -\frac{2}{n_{s}n_{t}}\sum_{i}^{n_{s}}\sum_{j}^{n_{t}}\omega_{i}^{s,n}\omega_{j}^{t,n}k\left(z_{i,m}^{s},z_{j,m}^{t}\right) \end{array}\right].$$

Therefore, the loss function of the overall model can be calculated as follows:

(20) $$\underset{f}{\min}J_{s}+\alpha J_{t}+\lambda J_{\mathrm{ALMMD}}\left(p,q\right),$$

where α is the weight parameter of the loss target domain and λ is the weight parameter applying to the transfer ALMMD loss. The specific process is shown in Algorithm 1.


**Algorithm 1** SACN training algorithm1: Initialize trainable parameters: Feature extractor parameters $f_{\theta }$, routing weight matrix $W^{\mathrm{route}}$, adaptive list of ALMMD $\left\{\alpha^{n}\right\}$, pose weight matrix $W^{\mathrm{pose}}$
2: Initialize invariance parameters: Weight parameters α and λ, training epochs number *t*, error margin ε
3: Input source domain data $D_{s}\left\{\left(x^{s},y^{s}\right)\right\}$, target domain data $D_{t}\left\{\left(x^{t},y^{t}\right)\right\}$
4: For *n* = 1, 2, 3, …, *t* do5:      Feature extractor: $u_{k}=f_{\theta }\left(x_{i}^{s}\right)$, $u_{l}=f_{\theta }\left(x_{j}^{t}\right)$
6:      PCaps generation: $u_{k}^{\mathrm{PCaps}}\leftarrow u_{k}$, $u_{l}^{\mathrm{PCaps}}\leftarrow u_{l}$
7:      DCaps generation: $c_{ij}=\mathrm{softmax}{\left(W_{i}^{\mathrm{route}}u_{i}\right)}_{j}$, $u_{k}^{\mathrm{DCaps}}\leftarrow u_{k}^{\mathrm{PCaps}}$, $u_{l}^{\mathrm{DCaps}}\leftarrow u_{l}^{\mathrm{PCaps}}$
8:      Forward propagation: $y_{k\_pre}^{s}=MLP\left(\left\|u_{k(1)}^{\mathrm{DCaps}}\right\|,\left\|u_{k(2)}^{\mathrm{DCaps}}\right\|,\ldots ,\left\|u_{k(n)}^{\mathrm{DCaps}}\right\|\right)$,$y_{l\_pre}^{t}=MLP\left(\left\|u_{l(1)}^{\mathrm{DCaps}}\right\|,\left\|u_{l(2)}^{\mathrm{DCaps}}\right\|,\ldots ,\left\|u_{l(n)}^{\mathrm{DCaps}}\right\|\right)$
9:      Back propagation: $Loss=J_{s}\left(y_{k\_pre}^{s},y_{k}^{s}\right)+\alpha J_{t}\left(y_{l\_pre}^{t},y_{l}^{t}\right)+\lambda J_{\mathrm{ALMMD}}\left(u_{k}^{\mathrm{DCaps}},u_{l}^{\mathrm{DCaps}}\right)$
10: End for11: Output: prediction probability $y_{pre}^{t}$



## 4. GIS partial Discharge Experiment

### 4.1. Source Domain Data Acquisition

**Figure 3:**
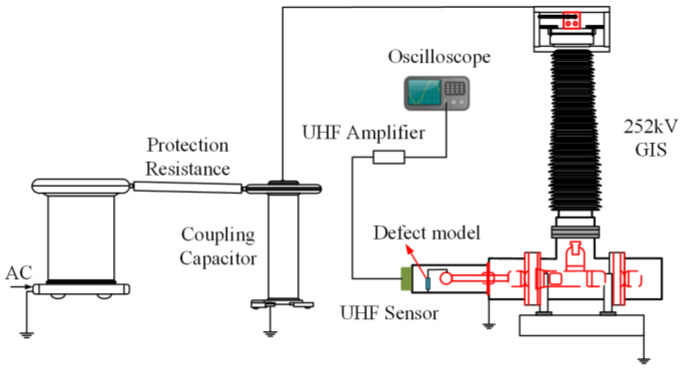
Experimental wiring schematic.

This study uses laboratory data as the source domain data. To build the source domain dataset, we built a 252-kV GIS PD experimental platform, as shown in Figure 3. The platform comprises a power source system, a GIS cavity, and a PD signal acquisition system. The power source system includes a PD power frequency test transformer and a voltage regulator. The rated capacity of the test transformer was 50 kVA, and the highest output voltage on the high-voltage side was 250 kV. The output voltage from the high-voltage side can be regulated in a range of 0–110 kV via voltage regulation of the low-voltage side. The total length of the GIS cavity is 7284 mm. Before the experiment began, the GIS cavity was vacuumed to remove gas impurities; then, the cavity was injected with SF_6_ until reaching a pressure level of 0.4 MPa. The PD signal acquisition procedure entailed an ultra-high-frequency (UHF) sensor receiving the high-frequency signals generated by PD in GIS. The signal was then amplified by a wide-band amplifier and the UHF signal was transmitted to an oscilloscope.

The key equipment parameters and models in the experimental system are given in the Table 2.

Four kinds of typical defects (tip discharge, free particle discharge, floating electrode discharge, and surface discharge) were simulated by artificial defect setting. (1) Tip discharge: A copper needle was installed on the high-voltage electrode to simulate the projection on the conductor surface. The length of the needle was 15 mm and the tip diameter was 0.5 mm. (2) Free particle discharge: A number of copper globes were peppered throughout the cavity as conductive metal particles. These globes can bounce as a result of the electrostatic force under AC voltage. (3) Floating electrode discharge: A 5 mm thick epoxy resin plate was deposited between the high-voltage electrode and the ground electrode. A copper plate was fastened to the epoxy resin plate at a height of 10 mm to keep the state of suspension. (4) Surface discharge: Copper wires (of 10 mm in length) were fixed on the surface of the epoxy resin. 

For each kind of defect, the test voltage was incrementally added to both ends of the test GIS in voltage steps of 2 kV as in the step-up voltage method. The voltage range was from 35 to 110 kV. PD occurs primarily at the initial voltage *U*_0_. If the discharge was sustainable, the PD signal was recorded and stored. The voltage was incremented in steps of 2 kV continuously when sustained discharge occurred. PD developed into flashover on the surface of the insulator as the test voltage increased. The corresponding voltage is the breakdown voltage *U_b_*. 

**Figure 4:**
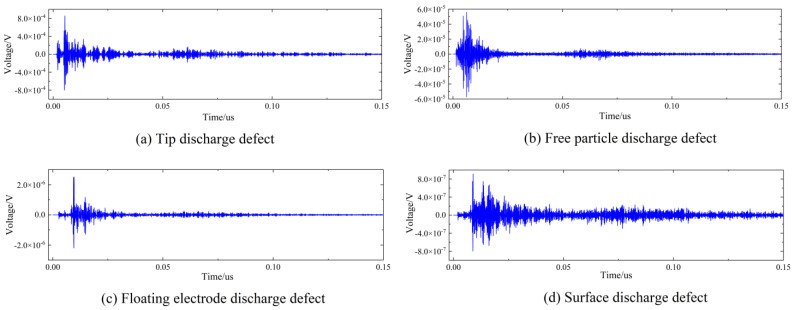
Waveform diagrams of four kinds of defects.

To obtain representative samples, two methods were used. The first method is repeating each test result 10 times and selecting the average value as the final result to avoid accidental errors of a single experiment. The second strategy involved choosing different positions of the simulated defect. Regarding surface discharge, the locations of the copper wires were positioned close to the high-voltage conductor, the center conductor, and the shell. Finally, after the experimental simulation of the four defects above, 1320 groups of samples (in which 330 groups of samples correspond to one kind of fault) were collected to establish the database of the source domain. The waveform diagrams of four kinds of defects are shown in Figure 4.

### 4.2. Target Domain Data Acquisition

The on-site defect samples were derived from years of historical maintenance data records of an electric power company in a chosen province. The historical raw data were affected by interference factors of the field operating environment. Therefore, after the process of labeling with the types of faults that occurred and uniformization to facilitate comparative and comprehensive analysis, the target domain dataset was built. Additionally, the initial data needed to be denoised because of the interference of environmental factors on site. The fast Fourier transform method was used for reducing the signal noise. A total of 320 groups of field samples were obtained, including 80 for tip charge defects, 40 for free particle discharge, 120 for surface discharge, and 80 for floating electrode discharge.

## 5. Result and Analysis

To demonstrate the superiority of the proposed model in PD diagnosis on small samples in the field, we conducted a comparative analysis from the feature extractors and domain adaptation methods. To demonstrate the excellent performance in the feature extraction of SR-CapsNet, we selected a CNN and CapsNet (dynamic routing algorithm) to compare the capability of PD diagnosis under the same number of layers. In addition, the superiority of the ALMMD subdomain adaptation was also verified by a comparison with other domain loss schemes such as MMD domain adaptation and LMMD subdomain adaptation. The feature extractors adopted in the above methods have the same structure as those of CapsNet. Finally, the superiority of the proposed method was verified by a comparison with existing methods.

The diagnosis network proposed was implemented on the PyTorch framework using the Python programming language. The network was implemented on a Windows 10 (64 bit) platform running on a PC with an i7-9750HF CPU, an NVIDIA RTX 3060 GPU, and a random-access memory of 16 GB.

The diagnosis accuracies for different feature extractors are shown in Table 3. It can be seen from Table 3 that the accuracies of SR-CapsNet were 11% and 12% higher than those of dynamic routing CapsNet on defects 0 and 1, respectively, which shows that self-routing further improves the diagnosis accuracy. The accuracies on defects 2 and 3 exhibited no improvement with dynamic routing. The performance of the CNN was significantly enhanced by CapsNet, which verifies that CapsNet compensates for the deficiency of ignoring the relationship between the local features and the relevant information hidden below by the CNN. The capsule layer, compared to the full-connection layer, can extract more features from the source domain to have initial recognition ability for almost all kinds of defects. As shown in Table 3, the feature distribution of the experimental data exhibit an obvious discrepancy with small samples in the field, so the model trained by the source domain directly is not suitable for on-site small samples.

To clearly display the significant advantage of the ALMMD subdomain adaptation, we compared it with other domain adaptation methods. The diagnosis accuracies of models with different domain adaptation methods are listed in Table 4. The table indicates that the MMD domain adaptation improves the overall accuracy of the PD diagnostic model using only CapsNet by 13.88% on small samples in the field. In addition, compared with MMD and LMMD, ALMMD improves the overall PD diagnostic accuracy by 11.12% and 5.5%, respectively.

**Figure 5:**
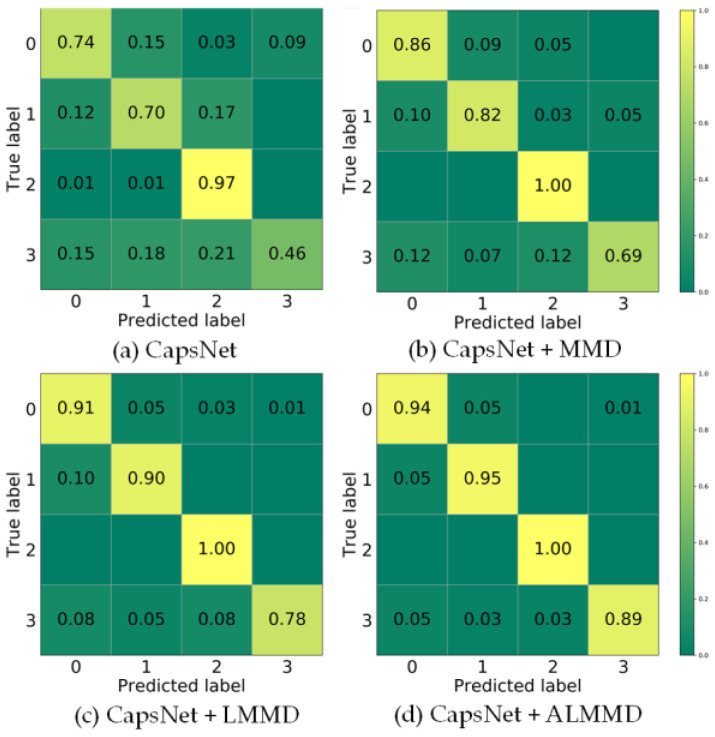
(**a**) Confusion matrix of CapsNet; (**b**) Confusion matrix of CapsNet with MMD domain adaptation; (**c**) Confusion matrix of CapsNet with LMMD subdomain adaptation; (**d**) Confusion matrix of CapsNet with ALMMD subdomain adaptation.

The confusion matrices of diagnosis performance on the different PD types utilizing no-transfer learning, MMD, LMMD, and ALMMD are shown as Figure 5, where 0, 1, 2, and 3 represent tip discharge, free particle discharge, floating electrode discharge, and surface discharge, respectively. As shown by confusion matrices (a) and (b), the addition of the MMD domain adaptation improved the classification accuracy notably, increasing the rate by 12%, 12%, 3%, and 23%, respectively. Moreover, the accuracy rate of defect 2 reached 100%. This demonstrates that the domain adaptation framework finds classification features that fit the target domain better and makes the discrimination effect of the four PD defect types more significant. As shown in confusion matrices (b) and (c), the accuracy of defects 0, 1, and 3 increased 5%, 8%, and 9%, respectively. This indicates that LMMD further improves the diagnostic accuracy of PD. As shown in confusion matrix (d), ALMMD increases the accuracy of defects 0, 1, and 3 by 3%, 5%, and 11%, respectively. This shows that the addition of adaptive coefficients can better measure the distance of each category sample and improve diagnostic accuracy. For defect 3, which has the lowest accuracy rate, both the discharge time and amplitude have great uncertainty. In addition, the features extracted from the surface discharge signal overlap with those of the other three types of defects. Therefore, defect 3 has a certain percentage of being misclassified as other defects. However, the accuracy of the ALMMD subdomain adaptation is closest to 90%.

**Figure 6:**
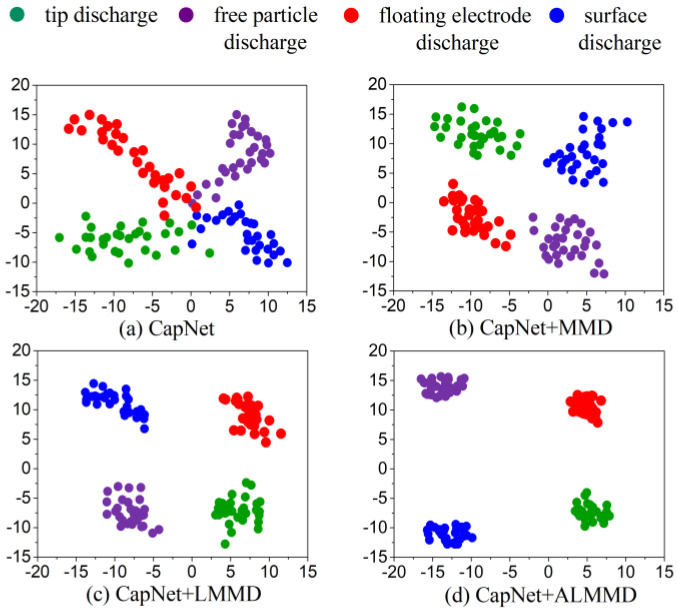
t-SNE results of different domain adaptation methods.

To visualize the advantages of ALMMD compared to other domain adaptation methods, t-distributed stochastic neighbor embedding (t-SNE) was used to obtain the two-dimensional visualization results in Figure 6. As shown in Figure 6a, different categories of subdomain boundaries are not well differentiated, and the distance between the samples of the same category is too large to be clustered together, which shows that the classification effect of only CapsNet is limited. The MMD domain adaptation in Figure 6b clearly reduced the confusion of the boundaries between each category, so the diagnosis accuracy increased greatly. Compared to MMD, LMMD in Figure 6c reduced the distance between samples in the same class, thereby further enlarging the distance between PD types. The distinguishing effect of ALMMD is better than that of the other three methods; its classification boundary of the four kinds of defects is the most remarkable, which demonstrates the superiority of feature extraction and high performance applied to the small-sample condition. It also shows that ALMMD not only matches the distribution at the global level but also matches the local distribution of different subdomains of the same category.

To evaluate the advantages of the proposed method, fine-tuning transfer learning (FTTL) [21], domain adversarial training (DAT) [22], and joint adaptation (JD) [23] were selected for comparison. The diagnostic accuracies of these methods are listed in Table 5. As shown in Table 5, FTTL had the lowest accuracy rate of only 82.5%, and its standard deviation was also the largest. JD had an accuracy of 84.73% and its standard deviation was smaller than that of FTTL and DAT. DAT aligned the global distribution match and further improved the average accuracy to 88.56%. The average accuracy of the SACN used in this study was the highest among all methods, reaching 93.75%. The relatively small standard deviation indicates its good robustness. Therefore, this indicates that the SACN can find more representative features at the subdomain level and has better diagnostic ability under the application conditions of small samples in the field.

## 6. Conclusions

We adopted an SACN for on-site PD defect diagnosis in GIS. For feature extraction, the self-routing improved CapsNet was adopted; this network can effectively use the relationship between features to reduce the loss of feature information and improve the efficiency of feature extraction. Compared with a CNN, the improvement in the feature extraction of CapsNet increases diagnosis accuracy by 36.12%. CapsNet introduces ALMMD subdomain adaptation, which achieves higher performance under the small-sample condition. By matching local distributions of different subdomains in the same category, ALMMD separates the classification boundary of different PD types more clearly. Compared with MMD and LMMD, ALMMD subdomain adaptation increases diagnosis accuracy by 11.12% and 5.5%, respectively. The superiority of the SACN in small-sample GIS PD diagnosis was verified by comparison with the current commonly used methods. However, the field data come from one data source, and multi-source result verification is required in the future. Additionally, the influence of the size of the target domain data on the model training and testing process is not validated directly; this aspect will be further studied in our next work.

## Figures and Tables

**Table 1:** Parameters of the feature extractor.

Layers	K-Size	Stride	Output Channels	Output Size
Conv1	116 × 1	8	32	128 × 32
MaxPool1	2 × 1	2	32	64 × 32
Conv2	34 × 1	2	32	16 × 32
MaxPool2	2 × 1	2	32	8 × (4) × 8
Capsule layer	4	-	1	4 × (8)

**Table 2:** Equipment parameters and models.

Equipment	Key Parameters
UHF sensor	Model: PDU-G2 Bandwidth: 300–1500 MHz Load impedance: 50Ω
Oscilloscope	Model: Agilent DSO9404 Analog bandwidth: 4 GHz Sampling rate: 20 GS/s
Amplifier	Gain: 40 dB

**Table 3:** Diagnostic accuracy of PD defects using different feature extractors.

Method	Diagnostic Accuracy				
	Tip (0)	Free Particle (1)	Floating Electrode (2)	Surface (3)	Overall (%)
CNN	0.97	0.07	0.15	0.10	32.63
CapsNet (dynamic routing)	0.63	0.58	0.95	0.47	64.38
CapsNet (self-routing)	0.74	0.70	0.97	0.46	68.75

**Table 4:** Diagnostic accuracy of different domain adaptation methods.

Method	Diagnostic Accuracy				
	Tip (0)	Free Particle (1)	Floating Electrode (2)	Surface (3)	Overall (%)
CapsNet	0.74	0.70	0.97	0.46	68.75
CapsNet + MMD	0.86	0.82	1.00	0.69	82.63
CapsNet + LMMD	0.91	0.90	1.00	0.78	88.25
CapsNet + ALMMD	0.94	0.95	1.00	0.89	93.75

**Table 5:** Diagnostic results of different methods.

Method	Average Diagnostic Accuracy (%)	Standard Deviation of Accuracy
FTTL	82.50	1.76
JD	84.73	0.93
DAT	88.56	1.19
SACN	93.75	0.67

## Data Availability

Data are unavailable due to privacy or ethical restrictions.
